# Rapid phagosome formation drives parasite control in subclinical *Leishmania braziliensis* infection

**DOI:** 10.3389/fimmu.2026.1837240

**Published:** 2026-06-05

**Authors:** Caic Figueiredo, Alan Rocha dos Santos, Camila Pimentel, Maurício T. Nascimento, Fabio Peixoto, Vítor Oliveira, Olivia Bacellar, Lucas P. Carvalho, Edgar M. Carvalho, Thiago M. Cardoso

**Affiliations:** 1 Laboratory of Clinical Research (LAPEC), Gonçalo Moniz Institute (IGM), Fiocruz, Salvador, Brazil; 2 Immunology Service, Complexo Hospitalar Universitário Prof. Edgard Santos, Federal University of Bahia, Salvador, Brazil; 3 National Institute of Science and Technology in Tropical Diseases (INCT-DT) Conselho Nacional de Desenvolvimento Científico e Tecnolígico (CNPq)/Ministério da Ciência, Tecnologia e Inovações (MCTI), Bahia, Brazil

**Keywords:** American tegumentary leishmaniasis, leishmania killing, phagosome formation, promastigotes, subclinical infection

## Abstract

**Introduction:**

Cutaneous leishmaniasis (CL) caused by *Leishmania braziliensis* displays a wide spectrum of clinical manifestations characterized by an inflammatory Th1 response that leads to the development of skin lesions. In endemic areas of *L. braziliensis* infection, individuals who present a delayed type of hypersensitivity reaction and/or IFN-γ production to soluble *Leishmania* antigen (SLA) without presenting or having a history of leishmaniasis are considered to have subclinical (SC) infection. SC subjects produce less IFN-γ and TNF-α as compared to CL subjects but control infection.

**Objective:**

This study proposed experiments to elucidate the involvement of phagosome formation in the control of intracellular parasites in macrophages (MØ) from SC individuals. The hypothesis of the study is that SC MØ present more quickly and effectively phagosome formation against intracellular parasites compared to CL MØ.

**Methodology:**

Monocyte-derived MØ were infected using calcein-labeled *L. braziliensis* promastigotes to determine parasite death and lysosome-associated membrane protein-1 (LAMP-1) (CD107a^+^) expression via flow cytometry to evaluate phagosome formation. The frequency of cells containing dead parasites and vesicle formation markers was determined via flow cytometry and immunofluorescence.

**Results:**

*L. braziliensis* killing was observed during the initial stages of infection (2 and 4 hours), maintaining this effective control even after 48 hours of infection. SC MØ exhibit higher expression of LAMP-1 during the initial events of infection as compared with cells from CL subjects and a lower number of viable parasites during the kinetics of infection.

**Conclusion:**

SC MØ can form phagosomes more quickly and for longer periods, providing effective parasite control, compared to CL MØ.

## 1 Introduction

Human cutaneous leishmaniasis (CL) caused by *Leishmania braziliensis* (*L.b*) is characterized by one or more well-limited ulcers with elevated borders (1). However, in areas of *L. braziliensis* transmission, approximately 20% of infected subjects have a positive *Leishmania* skin test (LST) and/or evidence of *in vitro* production of IFN-γ in supernatants of whole blood cultures stimulated with soluble *Leishmania* antigen (SLA) but do not develop CL and are considered as having a subclinical (SC) infection (2, 3).

The pathogenesis of CL is not completely understood, but pathology is associated with macrophage, NK, and CD8^+^ T-cell activation; high production of TNF, IL-1β, IL-17, and granzyme B; and NLRP-3 inflammasome activation (4–6). While peripheral blood mononuclear cells (PBMCs) from CL subjects produce high levels of pro-inflammatory cytokines after exposure to SLA, SC individuals produce lower levels of IFN-γ and of the other pro-inflammatory cytokines listed above but control infection without developing pathology. Regarding the role of specific cell populations in the defense against *Leishmania* in SC, it has been shown that while CD8^+^ T cells from CL subjects are predominantly cytotoxic and kill infected cells without killing the parasites, CD8^+^ T cells from SC subjects produce more IFN-γ and activate macrophages to kill *Leishmania* (7).

Neutrophils and macrophages are the first line of defense against *Leishmania* infection, and monocytes from SC subjects are less permissive to *L. braziliensis* and *Leishmania panamensis* infection (8–10). Macrophages are the main cells participating in *Leishmania* killing; however, despite the production of reactive oxygen species (ROS) and nitric oxide (NO), *L. braziliensis* survives in macrophages from CL subjects, contrasting with SC macrophages that, despite a low oxidative burst and low production of ROS and NO, effectively kill *Leishmania* (9a). However, the mechanisms by which individuals with SC achieve control over the infection are not yet understood. As the delay in phagolysosome maturation in *Leishmania*-infected cells decreases parasite killing (11), herein, we tested the hypothesis that SC macrophages present more rapid and effective phagosome/lysosome formation, which results in better *Leishmania* killing.

## 2 Materials and methods

This study was performed in the village of Corte de Pedra, an endemic area of *L. braziliensis* transmission located in the southeast of the state of Bahia, Brazil.

### 2.1 Case definition

SC individuals presented a positive LST and/or ability of PBMCs to produce IFN-γ upon *in vitro* exposure to SLA (soluble *Leishmania* antigen) and did not have evidence of current or previous CL. Patients with CL have a typical ulcer, and detection of *L. braziliensis* DNA was performed via PCR (12, 13); healthy subjects (HS) were from Salvador, a non-endemic area of CL, and did not produce IFN-γ after stimulation of whole blood cells with SLA.

### 2.2 Parasite culture and Calcein AM stain

The *Leishmania* isolate (MHOM/BR/2003/LTCP15344) characterized as *L.b* was obtained from a skin lesion of a CL patient after cultivation in biphasic medium Novy-MacNeal-Nicolle (NNN) medium. Promastigotes were cultured at 23 °C in Schneider’s Insect Medium (LGC Biotecnologia, Labtrade do Brasil, Cotia, São Paulo, Brazil) supplemented with 20% inactivated Fetal bovine sérum (FBS) (GIBCO BRL Life Technologies, Gaithersburg, MD, USA) and 1% Pen/Strep. Right before macrophage infection, promastigotes were labeled with calcein (following manufacturer protocol—Calcein AM Cell Viability Assay Kit ab270788, Abcam, Waltham, Massachusetts, United States); 1 × 10^6^ promastigotes/mL were resuspended to 390 μL in Phosphate Buffered Saline (PBS) 1×, and 10 μL of reconstituted calcein solution, ready to use (2 mM Calcein AM stock solution 1:5 by adding 200 μL PBS staining at a final concentration of 10 μM), was added. Promastigotes were incubated at 37 °C and 5% CO_2_ and protected from light for 30 minutes. Intracellular parasite death was evaluated via Fluorescence-Activated Cell Sorting (FACS) and immunofluorescence (Matthew A. 14–16) as well as by limiting dilution assay (LDA) (17**).**

### 2.3 Cell preparation and macrophage culture

PBMCs from SC patients, CL patients, and HSs were obtained via Ficoll-Hypaque gradient (GE Healthcare, Uppsala, Sweden). Monocytes were separated through negative selection using magnetic beads associated with specific antibodies (Dynabeads Untouched Human Monocytes Kit, Invitrogen, Thermo Fisher Scientific, Waltham, Massachusetts, United States). Monocytes were adjusted to a concentration of 1 × 10^6^ cells/mL in complete Roswell Park Memorial Institute (RPMI) 1640 medium and plated in 24-well cell culture plates (1 × 10^5^ cells/well) for 2 hours to achieve monocyte-derived macrophages (MØ). After this time, cell cultures were washed twice with complete RPMI medium supplemented with 10% heat-inactivated fetal calf serum, 100 U/mL penicillin, 100 mg/mL streptomycin, and 2 mL glutamine (Invitrogen, Carlsbad, CA, USA) to remove the non-adherent cells. The cells were maintained in culture for over 5 days, and the medium was replaced with complete RPMI after 48 hours.

At the same time, 5 × 10^4^ cells/well were plated in 4-well Lab-tek Chamber (Thermo Fisher Scientific) slide systems (as previously described for 24-well plate cultures) to perform intracellular parasite burden determination and immunofluorescence assay. Cell differentiation was monitored using the inverted optical microscope model AE31E (AE31E-Phase and Image-Pro^®^ Analysis Package-Motic).

### 2.4 Infection of macrophages

Calcein-labeled *L.b* promastigotes (in the stationary phase) were co-incubated with MØ (5:1 proportion) for 2 hours at 37 °C in 1 mL RPMI 1640. Non-ingested promastigotes were washed away with complete RPMI two times. Infected and uninfected MØ were cultured for 2, 4, 24, and 48 hours.

### 2.5 Macrophage phagosome formation

Infected MØ with previously calcein-labeled parasites were stained for lysosome-associated membrane protein-1 (LAMP-1) PE antibody clone H4A3 (RUO) 0.1 mg/mL, 0.5 µg per test (BD Biosciences, San Diego, CA, USA), to assess intracellular vesicle formation. Internalized parasites, fluorescing in the 517-nm spectrum, could also be detected via FACS as well as LAMP-1 expression to track the fusion of lysosomes with phagosomes (18–21). Samples of 2, 4, 24, and 48 hours’ kinetic points were acquired in a FACS Canto II flow cytometer (BD), and analyses were performed using the FACS Diva (BD) and FlowJo software (Tree Star, Ashland, OR, USA). Additionally, microscopy Lab-Tek chamber slides were used to perform immunofluorescence using labeled *L.b*, LAMP-1 PE antibody clone H4A3 (RUO) 0.1 mg/mL, at 0.5 µg/mL concentration (BD Biosciences, San Diego, CA, USA), and DAPI (Sigma-Aldrich, St. Louis, MO, United States). Images were captured using the confocal microscope Leica-SP8.

### 2.6 Evaluation of *L. braziliensis* killing

The frequency of infected macrophages and viable intracellular parasites was evaluated using Calcein AM expression, detected via cytometry and immunofluorescence analysis. Parasite burden was also evaluated via optical microscopy.

The viability of intracellular parasites was determined by the number of viable promastigotes of *L. braziliensis* using a limiting dilution assay. For this, cells infected with *L. braziliensis* were washed, and the medium was replaced with 0.5 mL of Schneider’s medium (Sigma-Aldrich) supplemented with 10% fetal calf serum to quantify the number of viable parasites. The plates were cultured at 26 °C for five additional days. Viable parasites were titrated, and the number of viable promastigotes was determined (17).

### 2.7 Statistical analysis

The Kruskal–Wallis with Dunn’s post-test was used to compare the frequency of infected MØ by calcein expression after cultures and co-expression of calcein and LAMP-1 on infected MØ and the number of viable parasites after a limited-dilution titration assay. The cut-off for statistical significance was set at p < 0.05, and results are shown as mean ± SD. Statistical analysis was performed using GraphPad Prism 5.0 (GraphPad Software, San Diego, CA, USA).

## 3 Results

### 3.1 Macrophages from subclinical individuals control the intracellular parasite burden

**Figure 1:**
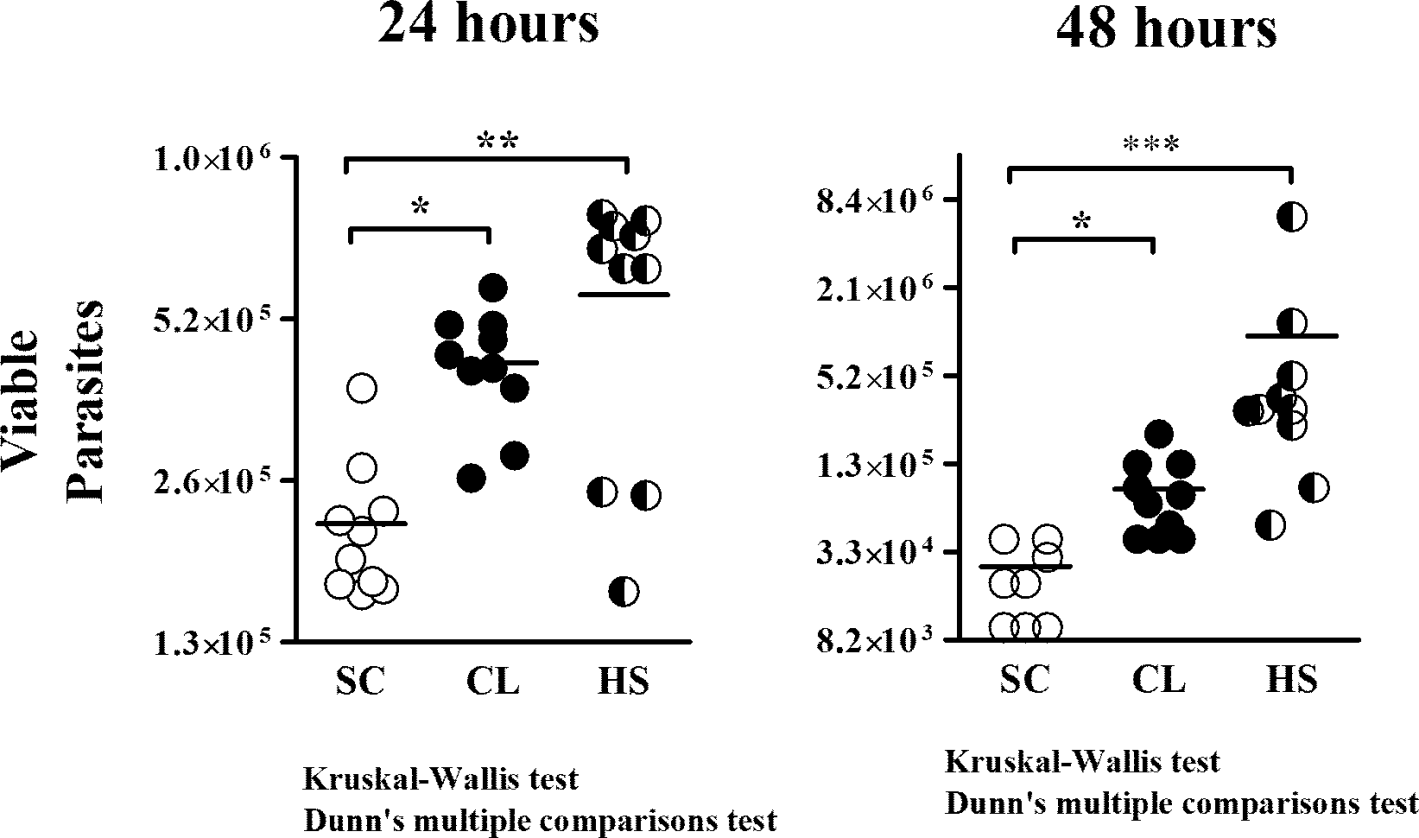
Number of viable parasites in macrophages from SC subjects and CL patients. The parasite-limiting titration assay shows that SC MØ efficiently kill parasites after 24 and 48 hours compared to CL MØ. Statistical comparisons were performed using the Kruskal–Wallis test and post-tested using Dunn’s multiple comparison test. p ≤ 0.05*/0.01**/0.001***. N = 10 per group. SC, subclinical; CL, cutaneous leishmaniasis; MØ, macrophages.

Studies have documented a lower frequency of infected MØ and a lower parasite load in MØ of SC individuals as compared with CL cells (9). Here, we compare in **Figure 1** the viability of *L. braziliensis* in MØ from SC subjects with CL using a limiting dilution assay. The frequency of parasites after 24 and 48 hours in SC cells (2.2 × 10^6^ ± 2.2 × 10^6^ and 2.6 × 10^5^ ± 2.2 × 10^5^ parasites) was lower than in CL cells (2.7 × 10^6^ ± 1 × 10^6^ and 8.8 × 10^5^ ± 5.5 × 10^5^, p ≤ 0.05 and p ≤ 0.01, respectively) and HS cells (5.8 × 10^6^ ± 2.6 × 10^6^ and 2 × 10^6^ ± 1 × 10^6^, p ≤ 0.01 and p ≤ 0.001, respectively).

### 3.2 Macrophages from subclinical individuals kill intracellular *L. braziliensis*

**Figure 2:**
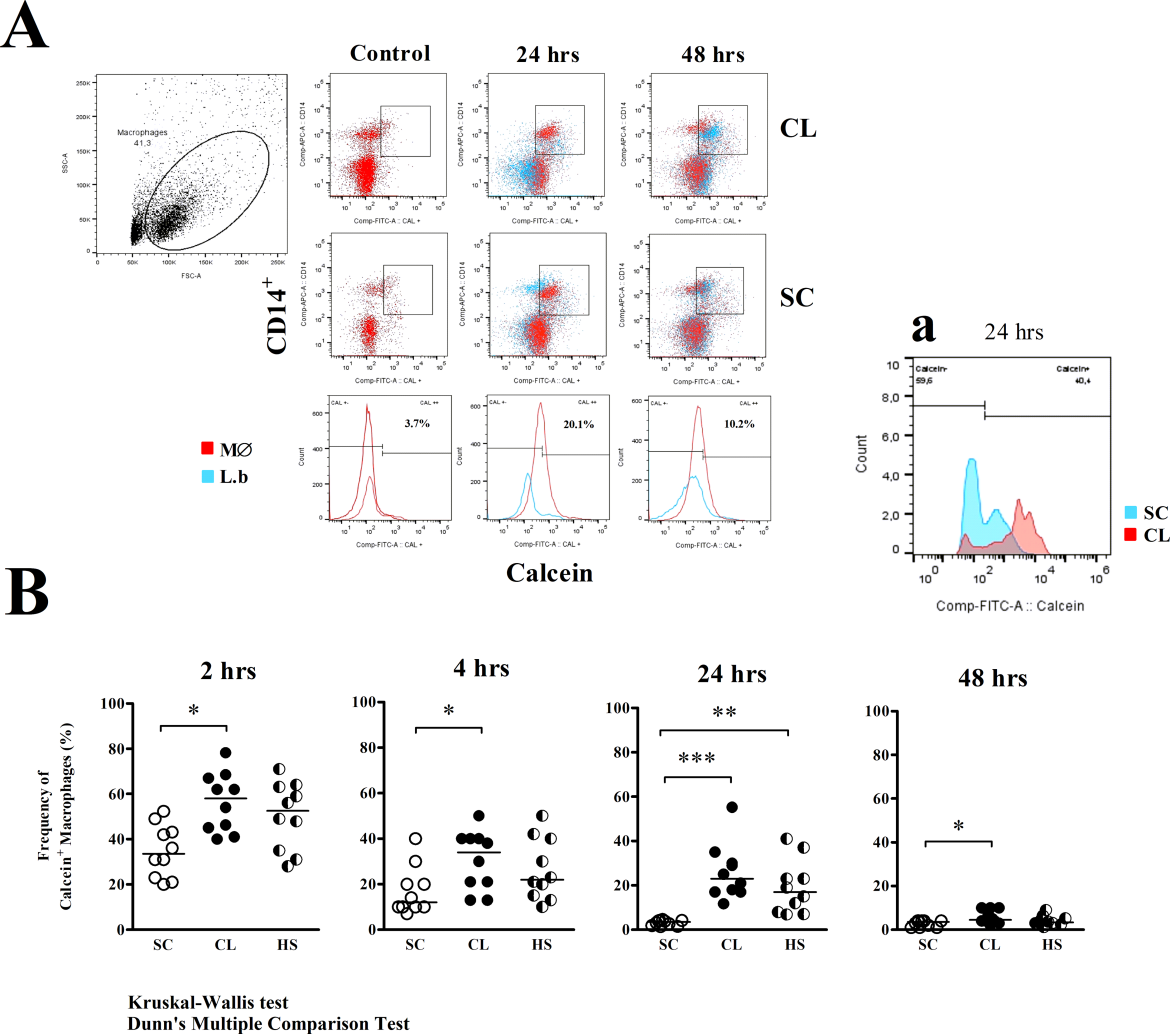
Intracellular killing of *Leishmania braziliensis* by macrophages from subclinical individuals and cutaneous leishmaniasis patients. **(A)** The gate strategy of FACS. of infected MØ with *L.b* labeled with Calcein AM selected by Forward Scatter (FSC) and Side Scatter (SSC) parameters shows that uninfected MØ (red dots) overlapped with infected MØ (cyan dots) during kinetics of 24 and 48 hours. The control is only uninfected MØ labeled with Allophycocyanin (APC)-conjugated CD14 and used to determine the auto-fluorescence detected at calcein wavelength (corresponding to 4.5% of background). (Aa) Histogram showing decreasing infected MØ with viable parasites in SC cells compared to CL cells after 24 hours of infection. **(B)** Frequency of calcein-labeled parasites in infected MØ from SC subjects was lower after 2, 4, 24, and 48 hours compared to those from CL subjects. Statistical comparisons were performed using the Kruskal–Wallis test and post-tested using Dunn’s multiple comparison test. p ≤ 0.05*/0.01**/0.001***. N = 10 per group. MØ, macrophages; *L.b*, *Leishmania braziliensis*; SC, subclinical; CL, cutaneous leishmaniasis.

The FACS strategy used to evaluate macrophage infection based on calcein expression is shown in **Figure 2A**. The co-expression of CD14 and calcein demonstrates the infected cell population at 2, 4, 24, and 48 hours of culture, and the loss of fluorescence of this marker allows us to trace the intracellular death of the parasite.

The frequency of infected MØ containing viable parasites after 2, 4, 24, and 48 hours of culture, tracked by calcein-labeled parasites, was lower in SC cells compared to CL and HS cells. Data shown in **Figure 2B**. After 2 hours, the frequency of infected cells in SC cultures was 34.8% ± 11.6% *versus* 56.4% ± 13.1% (p ≤ 0.05) in CL cultures; after 4 hours, 17.1% ± 10.6% in SC cultures and 30.6% ± 12.9% in CL cultures (p ≤ 0.05); after 24 hours, 3.1% ± 1.2% in SC cultures and 25.9% ± 12.5% in CL cultures (p ≤ 0.01 and p ≤ 0.001); and after 48 hours, 2.8% ± 1.4% in SC cultures and 5.9% ± 3.1% in CL cultures (p ≤ 0.05). Data shown in **Figure 2B**.

**Figure 3:**
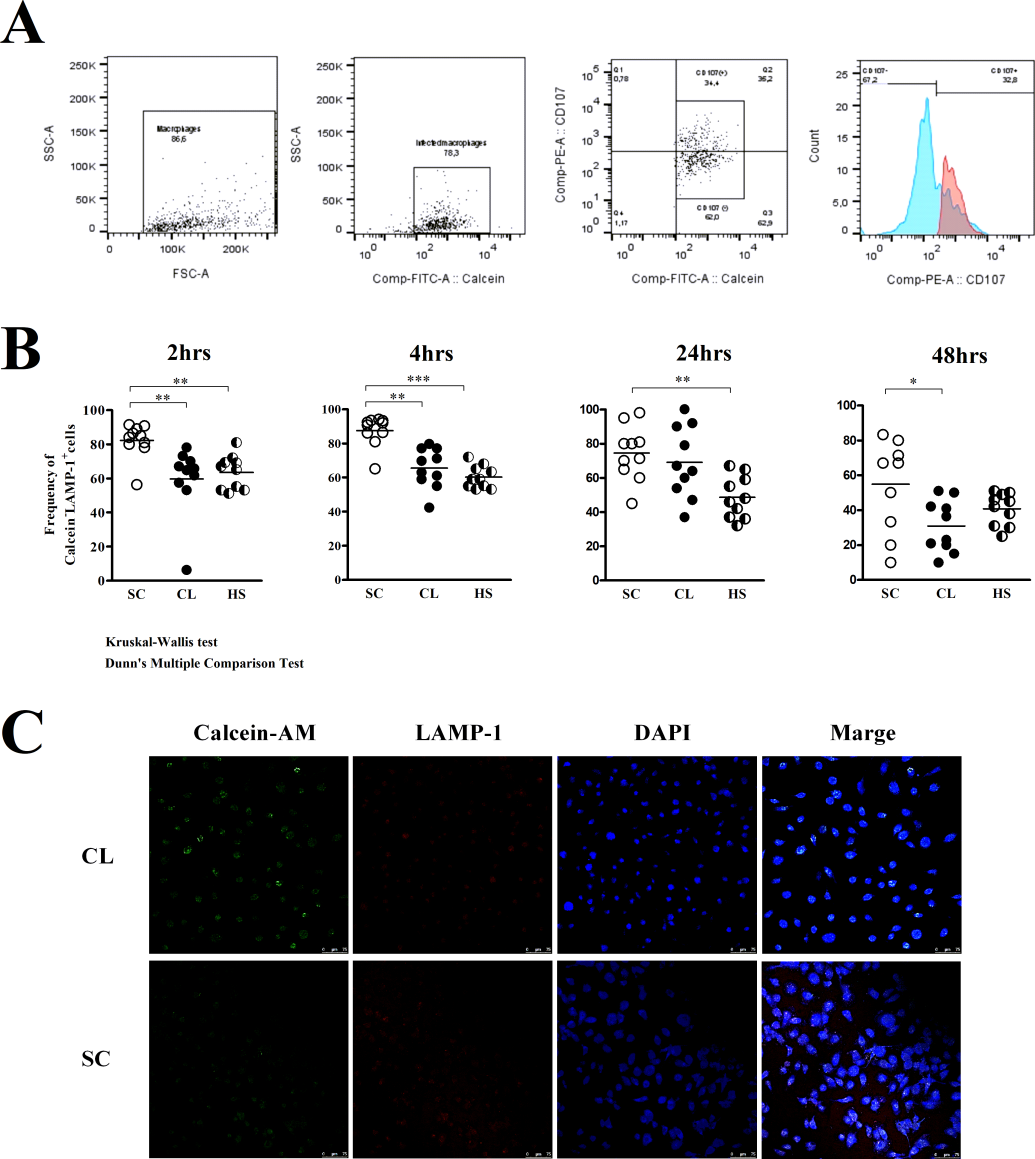
Phagosome formation and marker co-expression in macrophages. **(A)** Gate strategy of FACS of infected MØ with *L.b* labeled with Calcein AM selected by FSC and SSC parameters, showing the frequency of LAMP-1^(−)^ and LAMP-1^(+)^. **(B)** Frequency of infected MØ from SC and CL subjects shows a higher frequency of calcein^(−)^ and LAMP-1^(+)^ in SC compared to CL subjects after 2, 4, and 48 hours of infection. **(C)** SC- and CL-infected MØ immunofluorescence after 48 hours showing viable intracellular *L.b* labeled with Calcein AM, LAMP-1 PE, and DAPI. Herein, SC slide shows LAMP-1 expression and a few viable parasites beside CL-infected MØ. Statistical comparisons were performed using the Kruskal–Wallis test and post-tested using Dunn’s multiple comparison test. p ≤ 0.05*/0.01**/0.001***. N = 10 per group. MØ, macrophages; *L.b*, *Leishmania braziliensis*; SC, subclinical; CL, cutaneous leishmaniasis.

The LAMP-1 expression, a marker of phagosome formation, was determined via FACS (19, 20) and by immunofluorescence after calcein-labeled *L. braziliensis* infection at 2, 4, 24, and 48 hours. **Figure 3A** shows the strategy of analysis, first selecting calcein (CAL) positive and negative cells. The expression of LAMP-1 was used to determine phagosome formation on MØ containing viable (calcein^+^ cells) and unviable parasites (that lost Calcein AM fluorescence), shown hereafter at 24 and 48 hours as an example of cytometry analysis. **Figure 3A** shows the clearance of intracellular parasites observed in SC-infected MØ compared to CL after 24 hours.

The frequency of calcein^(−)^ and LAMP-1^(+)^ of MØ from SC subjects and CL is shown in **Figure 3B**. After 2 hours of infection, the frequency of cells expressing LAMP-1 and loss of calcein fluorescence in SC MØ (82.2% ± 10.1%) was higher (p ≤ 0.01) than in MØ from CL subjects (59.7% ± 20.1%). The higher frequency of cells calcein^(−)^ and LAMP^(+)^ continues to be observed up to 48 hours of infection (p ≤ 0.05).

**Figure 3C** shows immunofluorescence of the expression of LAMP-1 and calcein (viable intracellular parasites) in MØ from SC and CL cells after 48 hours of culture, demonstrating the persistence of phagosome formation even during the late stages of infection in SC MØ. The loss of calcein green fluorescence and presence of LAMP-1 staining cells, observed in SC cultures, is evidence of the ability to kill and the reduction of intracellular parasite load compared to those in CL cells.

## 4 Discussion

SC *L. braziliensis*-infected subjects usually have household contacts with CL patients who have the ability to control *Leishmania* and do not develop disease (22). However, the *Leishmania* killing by macrophages from these individuals is independent of ROS and NO (10, 23, 24). Phagosome formation is extremely important in the control and killing of intracellular parasites, including bacteria, fungi, and protozoa (25, 26; Christine 27, 28); in this study, in addition to determining phagosome formation, we introduced a novel technique for evaluating the death of amastigotes by the loss of the expression of Calcein AM, an indicator of the viability of phagocytosed parasites detected via flow cytometry (15, 16). Moreover, we measured the number of parasites using a limiting dilution assay after the transformation of amastigotes and promastigotes, and we documented that the number of viable promastigotes was lower in SC cells than in CL cells. Our observation that phagosome formation occurs early in SC cells and that parasite death is observed earlier in SC macrophages supports the idea that the rapid formation of the phagosome contributes to the greater capacity to kill parasites observed in SC subjects.

As the frequency of infected cells and parasite numbers is lower in MØ from SC subjects than in CL subjects after 2 hours of infection, it has been suggested that cells from SC subjects are less susceptible to *Leishmania* infection (22, 29). Here, we confirmed that parasite load in SC MØ is lower than that in CL MØ after 2 hours of infection, but we also observed a marked reduction of live parasites in infected SC MØ up to 24 hours after infection. Of note, the reduction in the percentage of infected cells between 4 and 24 hours was 82% in SC MØ and only 15% in CL MØ. In contrast, between 24 and 48 hours after infection, while the reduction in the percentage of infected cells was 10% in SC MØ, it was 77% in CL MØ. These data indicate that rather than a decrease in susceptibility to be infected by *L. braziliensis*, macrophages from SC individuals have a precocity ability of parasite killing.

The evaluation of phagosome formation in MØ from SC and CL individuals demonstrated that during the early stages of infection, a higher expression of LAMP-1 can already be observed in SC MØ compared to CL MØ, which was associated with a decrease in the number of viable promastigotes. The period from 2 to 24 hours appears to be the inflection point in which SC MØ phagosome formation more effectively controls parasite burden, as 48 hours after infection, the frequency of cells LAMP-1^(+)^ and calcein^(−)^ shows a 14.7% decrease, while in CL cells, there was an increase. After 24 hours, a decrease in phagosome formation was observed in SC cells (26%) and was even more pronounced in CL cells (55%).

Herein, a decrease in the number of amastigotes, evaluated via flow cytometry, microscopy, and limiting dilution assay combined with the expression of a parasite death marker observed at the same time of an increase in the expression of LAMP-1, reinforces the role that the earlier and persistent phagosome formation in SC-infected MØ plays an essential role in controlling *L. braziliensis* infection and indicates that a quicker formation of phagolysosomes is a characteristic of MØ from SC individuals that leads to more efficient *L. braziliensis* killing and parasite load control. Understanding how SC MØ kill intracellular *Leishmania* without causing tissue damage may lead to improved therapy against American tegumentary leishmaniasis (ATL) by increasing the host’s ability to kill *Leishmania*.

## Data Availability

The original contributions presented in the study are included in the article, further inquiries can be directed at the corresponding author.
